# Comparative Physiological and Transcriptome Profiles Uncover Salt Tolerance Mechanisms in Alfalfa

**DOI:** 10.3389/fpls.2022.931619

**Published:** 2022-06-09

**Authors:** Jiali Li, Maosen Ma, Yanmei Sun, Ping Lu, Haifan Shi, Zhenfei Guo, Haifeng Zhu

**Affiliations:** College of Grassland Science, Nanjing Agricultural University, Nanjing, China

**Keywords:** alfalfa, salt tolerance, transcriptome, ionic homeostasis, antioxidant defense system, unsaturated fatty acids

## Abstract

Salinity is a major limiting factor that affects crop production. Understanding of the mechanisms of plant salt tolerance is critical for improving crop yield on saline land. Alfalfa (*Medicago sativa* L.) is the most important forage crop, while its salt tolerance mechanisms are largely unknown. The physiological and transcriptomic responses in two contrasting salt tolerant cultivars to salinity stress were investigated in the present study. “Magnum Salt” showed higher salt tolerance than “Adrenalin,” with higher relative germination rate, survival rate, biomass and K^+^/Na^+^ ratio after salt treatment. Activities of antioxidant enzymes SOD, CAT and GR, and proline concentrations were upregulated to higher levels in roots and shoots in Magnum Salt than in Adrenalin after salinity stress, except for no difference in GR activity in shoots, and lower levels of O_2_^⋅–^ and H_2_O_2_ were accumulated in leaves. It was interesting to find that salinity caused a decrease in total unsaturated fatty acid in Adrenalin other than Magnum Salt, C18:2 was increased significantly after salinity in Magnum Salt, while it was unaltered in Adrenalin. High quality RNA sequencing (RNA-seq) data was obtained from samples of Magnum Salt and Adrenalin at different time points (0, 2, and 26 h). Generally, “phagosome,” “TCA cycle” and “oxidative phosphorylation” pathways were inhibited by salinity stress. Upregulated DEGs in Magnum Salt were specifically enriched in “fatty acid metabolism,” “MAPK signaling” and “hormone signal transduction” pathways. The DEGs involved in ionic homeostasis, reactive oxygen species (ROS) scavenging and fatty acid metabolism could partially explain the difference in salt tolerance between two cultivars. It is suggested that salt tolerance in alfalfa is associated with regulation of ionic homeostasis, antioxidative enzymes and fatty acid metabolism at both transcriptional and physiological level.

## Introduction

Salinity is one of the most important abiotic stresses affecting agricultural productions. Over 800 million hectares of arable lands are facing the problem of salinity in the world (Liu and Wang, 2021). Salt stress can cause growth inhibition even senescence and death in plants. Hyperosmosis is the primary transient effect of salinization to plants that lead to water absorption inhibition and dehydration. As exposure prolong, ionic equilibrium is interrupted with Na^+^ hyperaccumulation and K^+^ and Ca^2+^ influx hinder, followed by secondary injury such as photosynthesis inhibition, oxidative injury and metabolic disorder. Cultivation of salt-tolerant plants is a sustainable strategy to make better use of saline land, contributing to carbon neutral at mean time. The identification of salt tolerant plant germplasm resources and understanding their salt tolerance mechanisms are significant for new salt tolerant plant breeding and saline soil utilization.

Alfalfa (*Medicago sativa* L.) is the world’s most extensively cultivated forage legume. Improving salt tolerance is an important goal in alfalfa breeding. Different genotypes of alfalfa exhibit wide variations in salt tolerance. Yu et al. (2021) evaluated salt tolerance of 20 alfalfa cultivars at seedling stage and divided them into highly salt-tolerant, salt-tolerant, moderately salt-tolerant and salt-sensitive groups. Cornacchione and Suarez (2017) identified “SISA14” and “SW 8421S” populations as the most salt tolerant out of 15 alfalfa populations with high production in salt condition. In our research, salt-tolerance of 8 alfalfa cultivars was studied and one salt tolerant cultivar “Magnum Salt” and one salt sensitive cultivar “Adrenalin” were identified for further study.

Plants utilize multiple strategies to alleviate salt stress damage. Thus, salt tolerance is a complex trait controlled by multiple genes involving in salt perception, signaling transduction, osmotic regulation, ion transport, hormones synthesis, photosynthesis and metabolization (Deinlein et al., 2014). A few genes involved in salt resistance have been reported in alfalfa. For example, *MsCBL4* enhances calcium metabolism but not sodium transport in transgenic tobacco under salt stress (An et al., 2020); *MsPP2CA1* gene confers enhanced salt tolerance in *Arabidopsis*, which may due to osmolytes accumulation other than protecting the cell membrane from the damage by superoxide anion radicals (Guo et al., 2018). Besides, gene function of *MsGRP* (Long et al., 2013), *MsWRKY11* (Wang et al., 2018), *MsERF11* (Chen et al., 2012), *MsGME* (Ma et al., 2014), *MsRCI2A/B/C* (Li et al., 2021) and *MsZEP* (Zhang et al., 2016) in salt resistance have been reported.

RNA sequencing allows for analysis of the transcript levels as well as alternative splicing and novel transcript identification, showing great potential in tolerance mechanism studying and salt tolerant genes identification. Gruber et al. (2017) compared the transcriptome profiles of two salinity-tolerant alfalfa breeding populations and a more salinity-sensitive population, found that both saline-tolerant populations showed more substantial up-regulation in redox-related genes and B-ZIP transcripts. Time course full-length transcriptome database for alfalfa root tips under NaCl was constructed by Luo et al. (2019). Total of 8861 NaCl-regulated differentially expressed genes were identified, in which continuously up-regulated genes were mainly involved in “ion homeostasis,” “antiporter activity,” “trehalose biosynthetic process,” “thiamine pyrophosphate binding,” and “ethylene-activated signaling pathway.” By analyzing the transcriptome of salt tolerant “Halo” and salt intolerant “Vernal,” Bhattarai et al. (2021) found out that ion binding was the key molecular activity for salt tolerance in alfalfa. Furthermore, they identified 28 (leaf) and 31 (root) salt responsive candidate genes involved in transmembrane protein function, photosynthesis, carbohydrate metabolism, defense against oxidative damage, cell wall modification and protection against lipid peroxidation.

In our research, time course transcriptome database of roots from salt tolerant cultivar “Magnum Salt” and salt sensitive “Adrenalin” was conducted to understand the salt resistance mechanism of alfalfa. It was found that regulation of genes involved in K^+^, Na^+^, and Ca^2+^ homeostasis, reactive oxygen species (ROS) scavenging, as well as fatty acid metabolization, especially unsaturated fatty acid linoleic acid (C18:2) metabolization, might play important roles in salt resistance in alfalfa.

## Materials and Methods

### Evaluation of Germination Rate Under Salt Solution

A germination test was carried out using eight alfalfa cultivars, and 150 mM NaCl was used as salt treatment according to pre-experiments. Fifty plump seeds per petri dish were treated with 30 mL ddH_2_O or 150 mM NaCl solution with three replicates. The criterion of germination is the appearance of a 2 mm radicle protrusion. The relative germination was calculated as 100 × germination rate within 7 days in salt treatment group divided by germination rate in control group.

### Evaluation of Survival Rate Under Salt Treatment

Survival rates were evaluated according to Sun et al. (2020) with modification. The four-week old seedings planted in 50-pots tray were treated with 1 L Hoagland nutrient solution with or without 500 mM NaCl (pH 5.8) weekly for 3 weeks. The surviving plants with turgid and green leaves and dead plants were counted for calculation of survival rate after 1 week of recovery by washing NaCl solution with water.

### Evaluation of Biomass and K^+^/Na^+^/Ca^2+^ Concentration by Hydroponic Culture

Two-week old Magnum Salt and Adrenalin seedlings grown in soil with a mixture of pearlite, vermiculite and perlite (1:1:2) were transferred to a plate filled with 1/2 Hoagland nutrient solution (pH 5.8) for 1 week recovery growth. After that, seedlings were treated with 1/2 Hoagland nutrient solution containing 0 or 150 mM NaCl for 7 days, which was determined according to pre-experiments. Biomass were analyzed with 10 replicates, and K^+^/Na^+^/Ca^2+^ concentrations were measured as described by Dai et al. (2022).

### ROS, Antioxidant Enzyme Activities and Proline Content Analysis

Four-week old Magnum Salt and Adrenalin seedlings grown in soil (pearlite: vermiculite: perlite 1:1:2) were treated with ddH_2_O or 500 mM NaCl every 3 days, which was modified with reference to method of Dai et al. (2022). Six days later, DAB (3,3′-Diaminobenzidine) and NBT (ρ-nitro blue tetrazolium chloride) staining for H_2_O_2_ and O_2_^⋅–^ were performed as below. Leaves of the same position were collected followed by submerging into 0.1% DAB or NBT solution for 24 h. After decolorizing with 95% alcohol, photographs of the leaves were taken under an optical microscope. Antioxidant enzyme activity and free proline content were analyzed according to Zhuo et al. (2018).

### Fatty Acids Content Analysis

Roots from seedlings treated with 500 mM NaCl solution for 3 and 6 days were collected for fatty acid content analysis. Gas chromatographic analysis was used and the procedure was as described previous (Huang et al., 2017). DBI (Double-bond index) was used to measure the degree of lipid total unsaturation. DBI = [1 × (% 18:1) + 2 × (% 18:2) + 3 × (% 18:3)]/100, as described by De Vos et al. (1993).

### RNA Extraction, Library Construction, and Sequencing

Two-week old seedlings grown in soil with a mixture of pearlite, vermiculite and perlite (1:1:2) were transferred to a plate filled with 1/2 Hoagland nutrient solution (pH 5.8) for 1 week recovery growth. After that, seedlings were treated with 1/2 Hoagland nutrient solution containing 150 mM NaCl, roots were sampled at 0, 2, and 26 h. Total RNA of roots were extracted using a RNAprep pure Plant Kit (Tiangen Inc., Beijing, China). mRNAs were purified using Dynabeads Oligo(dT)_25_ (Life Technologies). Ribo-Zero™ Magnetic Kit (Epicentre) was used to remove rRNA. The derived mRNAs were fragmented and reverse transcribed into cDNAs with random hexamer. The cDNAs were purified and ligated to adaptors for Illumina paired-end sequencing. The cDNA library was sequenced using the Illumina HiSeq™4000 system by Gene Denovo Biotechnology Co. (Guangzhou, China). The sequencing data has all been archived in the NCBI Sequence Read Archive (SRA) database under accession number PRJNA830977.

### Unigene *de novo* Assembly and Annotation

Raw reads were initially cleaned by removing adaptor sequences and low quality reads to get clean reads. The Trinity program was carried out to assemble the clean reads to obtain nonredundant unigenes. The assembly unigenes functional annotations were obtained using NCBI nucleotide sequence database (Nt), nonredundant protein database, Swiss-Prot database, Clusters of Orthologous Groups of proteins (COG) database, Gene Ontology (GO) annotation and Kyoto Encyclopedia of Genes and Genomes (KEGG) annotation database.

### Identification of Differentially Expressed Genes

Differentially expressed genes (DEGs) was analyzed by a DESeq R package^1^ with the thresholds of false discovery rate (FDR) < 0.05 and | Log2Fold-Change| > 4. Then DEGs GO enrichment and KEGG analysis were performed with agriGO 2.0^2^ and KOBAS 3.0^3^, respectively.

### Differentially Expressed Genes Temporal Expression Patterns Analysis

To achieve the temporal expression patterns, the DEGs of at 2 and 26 h after salt treatment were analyzed with the Short Time-series Expression Miner (STEM) software (v1.3.11; Ernst and Bar-Joseph, 2006). The potential function annotations and enrichments of DEGs identified in STEM analysis were explored with KEGG analysis.

### Heatmap Analysis

FPKM was used for representing the expression abundance of genes. The clustered heatmap was portrayed after normalized with zero-to-one scale method using the TBtools software (Chen et al., 2020).

### Quantitative Real-Time PCR Analysis

cDNA synthesis was performed with HiScript III RT SuperMix for qPCR (+gDNA wiper) reagent kit with gDNA Eraser (Vazyme, China). qRT-PCR reaction was performed following the instructions of ChamQ Universal SYBR qPCR Master Mix (Vazyme, China). All primer sequences are shown in Supplementary Table 5. The relative gene expression level was calculated with 2^–ΔΔCt^ method.

### Statistical Analysis

**FIGURE 1:**
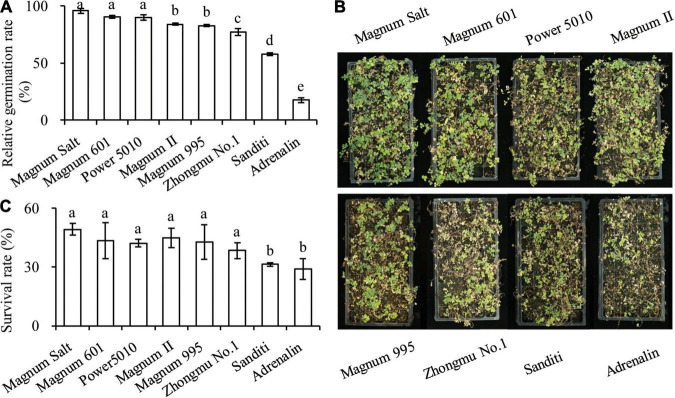
Evaluation of salt tolerance of 8 alfalfa cultivars by analyzing relative germination rate and survival rate under salt condition. Alfalfa seeds were treated with 0 or 150 mM NaCl solution, and relative germination rate **(A)** were calculated within 7 days. Four-week old seedlings treated with Hoagland nutrient solution with 500 mM NaCl (pH 5.8) weekly. Three weeks later, photographs **(B)** were taken and survival rates **(C)** were analyzed. Values are mean ± SE (*n* = 3 replicates). Different letters (*P* < 0.05, one-way ANOVA) compare different cultivars.

All data were subjected to analysis of variances according to the model for completely randomized design using an SPSS program (SPSS Inc., Chicago, United States). Significant differences among means of cultivars in Figure 1 were evaluated by one-way ANOVA at 0.05 probability level. Significant differences among means of both treatments and cultivars were evaluated by LSD test at 0.05 probability level.

## Results

### Identification of Salt-Tolerant and Salt-Sensitive Cultivars

Salt tolerance of eight commonly planted cultivars was evaluated based on relative germination rate and survival rate in response to salt treatment. The relative germination rate decreased in “Magnum Salt,” “Magnum 601,” “Magnum II,” “Magnum 5010,” “Zhongmu No.1,” “Sanditi,” and “Adrenalin” sequentially (Figure 1A). “Magnum Salt,” “Magnum 601,” “Magnum II,” “Magnum 5010,” and “Zhongmu No.1” had significantly higher survival rate than “Sanditi” and “Adrenalin” after the seedling were irrigated with 500 mM NaCl solution for 3 weeks (Figures 1B,C). The data indicated that “Magnum Salt” and “Adrenalin” showed the highest and lowest salt tolerance, respectively.

**FIGURE 2:**
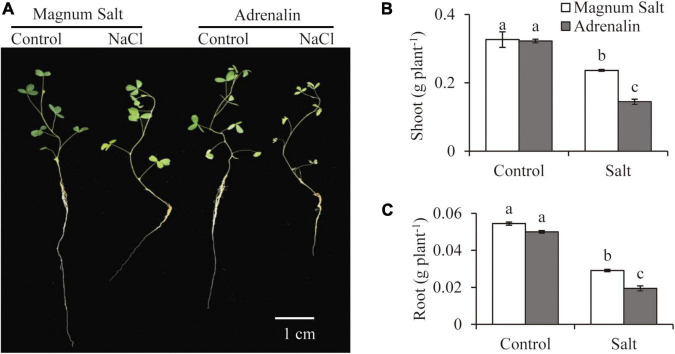
Effect of salt treatment on the biomass of Magnum Salt and Adrenalin. Three-week old seedlings were treated with 1/2 Hoagland solution containing 0 or 150 mM NaCl for 1 week. Photographs **(A)** were taken and biomass of shoot **(B)** and root **(C)** were analyzed. Values are mean ± SE (*n* = 10 replicates). Different letters indicate the significant differences (*P* < 0.05, LSD) between two cultivars or treatments.

Growth performance of Magnum Salt and Adrenalin was further evaluated by hydroponic culture experiment (Figure 2). Treatment with 150 mM NaCl caused obvious inhibition of root growth and yellowing of leaves in both cultivars (Figure 2A). Dry weight of roots or shoots showed no significant difference between Magnum Salt and Adrenalin under control condition (Figures 2B,C). It was decreased after salt treatment in both cultivars, while that was higher in Magnum Salt than that in Adrenalin (Figures 2B,C).

### Analysis of Na^+^, K^+^ and Ca^2+^ Concentrations

**FIGURE 3:**
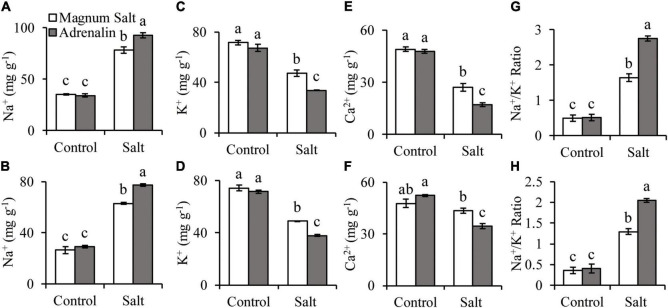
Effect of salt treatment on the Na^+^, K^+^ and Ca^2+^ accumulation in Magnum Salt and Adrenalin. Three-week old seedlings were treated with 1/2 Hoagland solution containing 0 or 150 mM NaCl for 1 week. Na^+^, K^+^, Ca^2+^ content and Na^+^/K^+^ ratio in roots **(A,C,E,G)** and shoots **(B,D,F,H)** were analyzed. Values are mean ± SE (*n* = 3 replicates). Different letters indicate the significant differences (*P* < 0.05, LSD) between two cultivars or treatments.

The Na^+^ concentration was increased in both roots and shoots after 7 d of salt treatment, with higher level in Adrenalin than in Magnum Salt (Figures 3A,B). K^+^ and Ca^2+^ concentrations were decreased in both cultivars, with higher levels in Magnum Salt than in Adrenalin after salt treatment (Figures 3C–F). As a consequence, the Na^+^/K^+^ ratio in roots and shoots were higher in Adrenalin than in Magnum Salt (Figures 3G,H).

### Analysis of Antioxidants, Proline and ROS Accumulation

**FIGURE 4:**
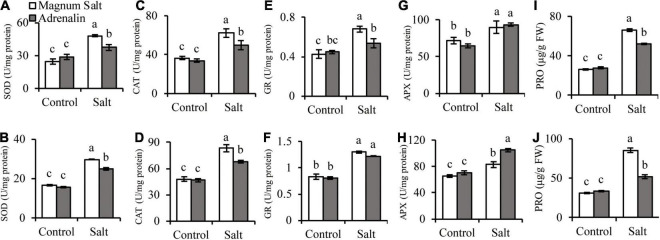
Effect of salt treatment on the antioxidant enzyme activities and proline content in Magnum Salt and Adrenalin. Four-week old seedlings grown in soil with a mixture of pearlite, vermiculite and perlite (1:1:2) were treated with 0 or 500 mM NaCl for 6 days. SOD, CAT, GR, and APX activities and proline contents were analyzed in both root **(A,C,E,G,I)** and shoot **(B,D,F,H,J)**. Values are mean ± SE (*n* = 3 replicates). Different letters indicate the significant differences (*P* < 0.05, LSD) between two cultivars or treatments.

Antioxidant enzyme activities and proline concentrations were measured after plants were treated with NaCl. SOD, CAT, GR, and APX activities showed no difference between two cultivars under control condition. SOD, CAT, and GR were increased after salt treatment, and higher levels were observed in roots and shoots in Magnum Salt than that in Adrenalin, except for no difference in GR activity in shoots (Figures 4A–F). APX activity was increased in both cultivars after salt treatment and showed no difference in roots, but higher level was maintained in shoots in Adrenalin than in Magnum Salt (Figures 4G,H). No significant difference in proline concentration was observed between two cultivars under control condition. It was increased greatly in roots and shoots in both cultivars, while higher levels were maintained in both roots and shoots in Magnum Salt than in Adrenalin after salt treatment (Figures 4I,J).

**FIGURE 5:**
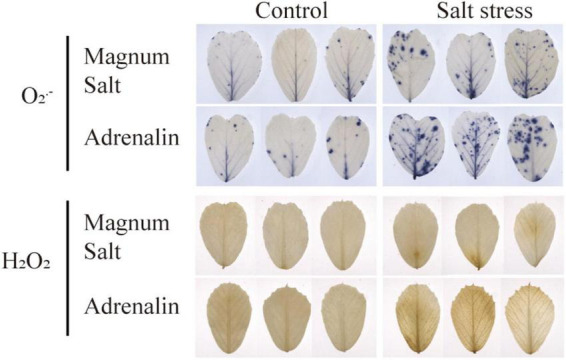
Effect of salt treatment on the ROS content in Magnum Salt and Adrenalin. Four-week old seedlings grown in soil with a mixture of pearlite, vermiculite and perlite (1:1:2) were treated with 0 or 500 mM NaCl for 6 days. Leaves were sampled for NBT and DAB staining.

Leaves were sampled for detection of ROS accumulation using NBT and DAB staining. Compared to the control leaves, O_2_^⋅–^ and H_2_O_2_ accumulation was observed in leaves in both cultivar after salt treatment, while lower levels were maintained in Magnum Salt than in Adrenalin (Figure 5).

### Analysis of Fatty Acids Content

**FIGURE 6:**
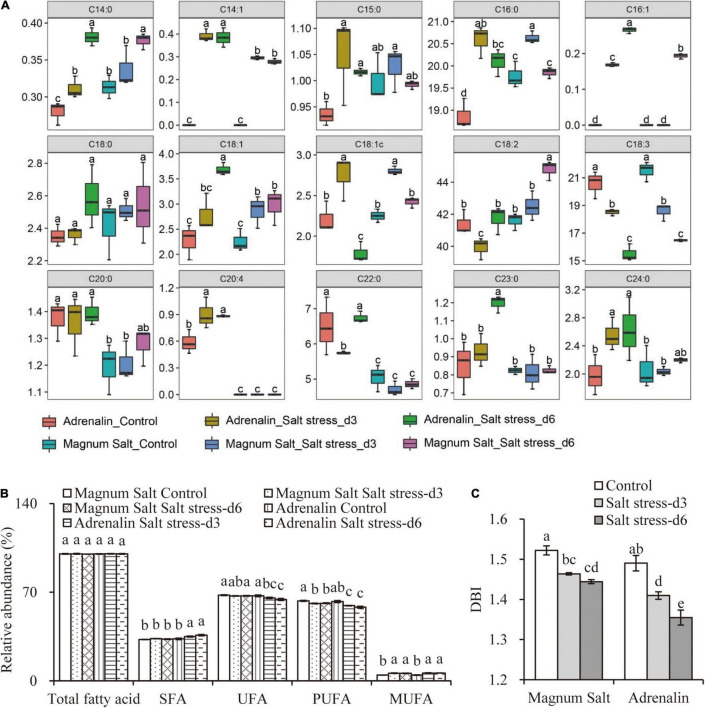
Effect of salt treatment to the Fatty acids content in Magnum Salt and Adrenalin. Four-week old seedlings treated with 0 or 500 mM NaCl. At 0, 3 and 6 days, roots were sampled for fatty acids content analysis **(A)**. The upper and lower edges of the box in the boxplot represent the maximum and minimum values, respectively, and the horizontal line in the middle represents the median values. Relative abundance of saturated fatty acids (SFA), unsaturated fatty acid (UFA), monounsaturated fatty acids (MUFA), polyunsaturated fatty acid (PUFA); **(B)** and double bond index (DBI; number of double bonds per mole) **(C)** were calculated. Values are mean ± SE (*n* = 3 replicates). Different letters indicate the significant differences (*P* < 0.05, LSD) between two cultivars or treatments.

Nearly 90% of total fatty acids were C16 and C18 fatty acids in alfalfa roots under control condition, among them were major C18:2 (40%), C18:3 (20%) and C16:0 (20%). The portion of C18:2 increased significantly after 6 d of salt stress in Magnum Salt, but it was slightly decreased after 3 d of salt treatment, followed by a recovery at 6 d in Adrenalin. C18:3 content continuously decreased after salinity treatment, with no significant difference between two cultivars. C16:0 fatty acid was higher in Magnum Salt than in Adrenalin under control condition, and it showed no significant difference between two cultivars under salt stress (Figure 6A). Both saturated fatty acid (SFA) and unsaturated fatty acid (UFA) abundance were not altered in Magnum Salt after salinity treatment, while SFA was slightly increased but UFA was decreased in Adrenalin (Figure 6B). The monounsaturated fatty acids (MUFA) abundance was increased under salt stress, while polyunsaturated fatty acid (PUFA) was decreased, with lower level in Adrenalin. Double bond index (DBI; number of double bonds per mole) showed no difference between two cultivars under control condition, but it was decreased after salinity treatment with higher level in Magnum Salt than in Adrenalin (Figure 6C).

### Identification and Functional Annotation of Differentially Expressed Genes

In order to study the expression profiles of genes in response to salinity, cDNA libraries representing samples of two cultivars (S-Adrenalin; T-Magnum Salt) at different time points (0, 2, and 26 h) were constructed for high-throughput RNA-Seq. A total of 740,274,514 raw reads were obtained (Supplementary Table 1).

**FIGURE 7:**
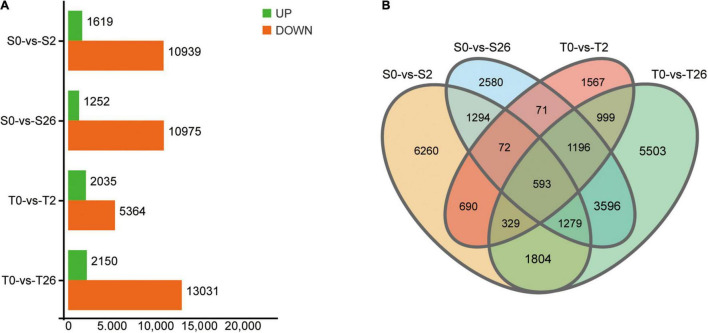
Differentially expressed genes (DEGs) in Magnum Salt (T) and Adrenalin (S) under salt stress. **(A)** Number of upregulated and downregulated DEGs under salt stress. **(B)** Venn diagram analysis of the DEGs in different comparison groups of S0-vs-S2, S0-vs-S26, T0-vs-T2 and T0-vs-T26.

A total of 735,954,286 clean reads remained after removing the adaptor sequences and low-quality sequences. Then, 79,712 unigenes with average GC content of 40.71% and N50 value of 1260 bp were obtained (Supplementary Table 1). By searching against four databases (Nr, Swiss-Prot, KOG and KEGG), 53171 unigenes were annotated (Supplementary Table 2). A total of 47365 DEGs matching the thresholds of false discovery rate (FDR) < 0.05 and | Log2Fold-Change| > 4 were identified (Figure 7). At 2 h, 12558 and 7399 DEGs were detected in Adrenalin and Magnum Salt, with 1684 DEGs in common. After 26 h treatment, 12227 and 15181 DEGs were identified in Adrenalin and Magnum Salt, with 6664 DEGs in common. There were 1684 and 6664 genes that differently expressed in both cultivars at 2 and 26 h.

### Differentially Expressed Genes Temporal Expression Patterns Analysis

Temporal expression patterns of the DEGs at 2 and 26 h after salt treatment were analyzed with the Short Time-series Expression Miner (STEM) software (v1.3.11). Eight different profiles of DEGs identified in STEM analysis were explored and enriched with KEGG analysis (Supplementary Table 3). Profile 0, exhibited a consistent decreasing trend, contains 6464 DEGs enriched in “phagosome,” “endocytosis,” “TCA cycle,” “carbon metabolism,” “glyoxylate and dicarboxylate metabolism,” “steroid biosynthesis,” “tryptophan metabolism,” “oxidative phosphorylation,” “arginine and proline metabolism” in Magnum Salt, and 2845 DEGs enriched in “phagosome,” “oxidative phosphorylation,” “isoquinoline alkaloid biosynthesis,” “cysteine and methionine metabolism,” “ribosome biogenesis,” “TCA cycle,” “steroid biosynthesis,” “cutin, suberine and wax biosynthesis,” “sesquiterpenoid and triterpenoid biosynthesis” in Adrenalin. Profile 7 exhibited an opposite trend. In Magnum Salt, 912 continuously upregulated DEGs were contained in Profile 7, and were enriched mainly in “linoleic acid metabolism,” “MAPK signaling pathway” and “hormone signal transduction” and so on. However, 393 DEGs in Adrenalin were contained in Profile 7, and were enriched mainly in “pyruvate metabolism,” “endocytosis,” “glycolysis.” Notably, “phagosome,” “TCA cycle” and “oxidative phosphorylation” pathways were frequently present in enrichment results of profile 0, 1, and 3, indicating that vesicle transport and aerobic metabolism were inhibited under salinity stress. The DEGs in profile 4, 6, and 7 in Magnum Salt were frequently enriched in “fatty acid metabolism,” “MAPK signaling pathway” and “hormone signal transduction” pathways, implying that fatty acid metabolization and signal transduction played an important role in coping with salt in alfalfa.

### Differentially Expressed Genes Involved in Na^+^, K^+^ and Ca^2+^ Transport, ROS Scavenging, and Fatty Acid Metabolization

**FIGURE 8:**
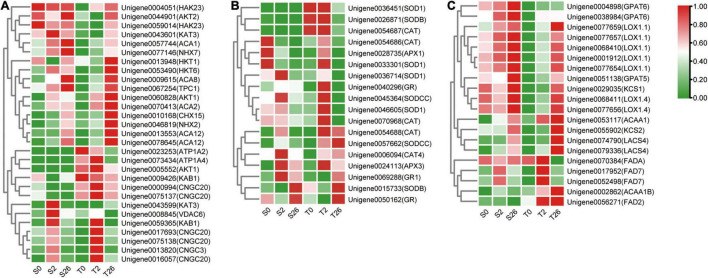
Heatmap of the relative expression of the DEGs involved in Na^+^, K^+^ and Ca^2+^ transport **(A)**, ROS-scavenging **(B)** and fatty acid metabolization **(C)** in Magnum Salt and Adrenalin under salt stress. FPKM was used for representing the expression abundance of genes. The clustered heatmap was portrayed after normalized with zero-to-one scale method using the TBtools software.

DEGs involved in K^+^/Na^+^/Ca^2+^ transport were investigated in our research (Figure 8A and Supplementary Table 4). The levels of *AKT1*, *NHX2*, *HKT1* and *HKT6* that involved in sodium/potassium homeostasis were higher in Magnum Salt after 26 h salinity, nevertheless, *NHX7*, *AKT2*, *HAK23* and *KAT3* always had higher expression in Adrenalin. Six unigenes annotated as *CNGC3* and *CNGC20* were expressed at higher levels in Magnum Salt than in Adrenalin at 2 h. At 26 h, *ACA2*, *ACA8* and *ACA12* involved in Ca^2+^ influx or efflux pathways were upregulated and both *ACA2* and *ACA12* were expressed at higher levels in Magnum Salt.

Differentially expressed genes encoding SOD, CAT, APX and GR were analyzed. Most of them were upregulated after 2 or 26 h of salt treatment in Magnum Salt, while less genes were upregulated in Adrenalin (Figure 8B and Supplementary Table 5).

DEGs involved in fatty acid metabolization pathway were highly enriched in profile 7 in Magnum Salt (Supplementary Table 3). *KCS2*, *ACAA1*, *ACAA1B*, *LACS4*, *FAD2* and *FAD7* were upregulated to higher levels in Magnum Salt by NaCl. Nevertheless, the expression levels of *GPAT5* and *GPAT6* increased more strongly in Adrenalin following salt stress (Figure 8C and Supplementary Table 6). The transcript levels of *KCS1*, *LOX1.1* and *LOX1.4* were induced by salinity stress, but they were expressed at higher levels in Adrenalin.

### RNA-Seq Results Validation With qRT-PCR Analysis

**FIGURE 9:**
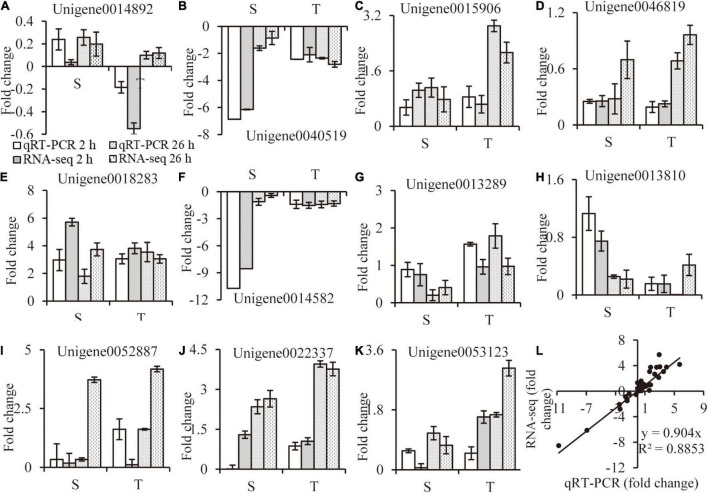
RNA-seq data validation. Random selected genes with different expression patterns were analyzed by qRT-PCR **(A–K)**, correlation between qRT-PCR and RNA-seq data are calculated **(L)**. Values are mean ± SE (*n* = 3 replicates).

The relative folder change of eleven randomly selected DEGs were analyzed by qRT-PCR to verify the RNA-Seq results. Primers were listed in Supplementary Table 7. A high consistency was detected for these genes between qRT-PCR and RNA-seq results (Figures 9A–K). As shown in Figure 9L, a strong positive correlation (*R*^2^ = 0.8917) was obtained by a linear regression analysis, suggesting that our transcriptome data were reliable.

## Discussion

By analyzing the germination rates, survival rates and biomass under salt treatment, “Magnum Salt” and “Adrenalin” were identified as salt tolerant and salt sensitive cultivar, respectively (Figures 1, 2). Both physiological and transcriptional responses of these two cultivars to salinity stress were investigated. It was found that regulation of ionic homeostasis, fatty acid metabolization and antioxidative system played important roles in salt tolerance in alfalfa.

### Ion Homeostasis

Maintaining ion homeostasis, especially a low cytosolic Na^+^/K^+^ ratio, is an important adaptive trait of salt-tolerant plants, which was also observed in salt-tolerant cultivar Magnum Salt in our experiments (Figures 3G,H). The transporters or channels involved in Na^+^ efflux and K^+^ uptake play important roles in salt resistance. NHX-type cation/H^+^ transporters in plants have been shown to mediate Na^+^(K^+^)/H^+^ exchange for salinity tolerance and K^+^ homoeostasis, providing an important strategy for ionic homeostasis in plants under saline conditions (Apse et al., 1999; Zeng et al., 2018). There were two salt-upregulated *NHX* genes identified in DEGs, *NHX2* and *NHX7*. *NHX2* expression abundance was about three times higher than *NHX7* in all samples collected in our research (Supplementary Table 4). Notably, *NHX2* increased greater in Magnum Salt than in Adrenalin. High-affinity K^+^ channel (HKT) and a low-affinity K^+^ channel Arabidopsis K^+^ Transporter1 (AKT1) were reported to mediate K^+^/Na^+^ influx (Ali et al., 2021, Wang et al., 2021). *KAT3* encoded a member of the shaker family of voltage-gated potassium channel subunits and responded to low potassium stress in Arabidopsis, however, its function in salinity response had not been reported (Wang et al., 2016). The expression of *AKT1*, *HKT1* and *HKT6* were significantly higher in Magnum Salt after 26 h salinity, while genes encoding HKT23 or KAT3 were highly expressed in Adrenalin (Figure 8A).

Plant response to salt is closely linked to the calcium (Ca^2+^) channels as well as Ca^2+^ sensing and signaling (Seifikalhor et al., 2019, Feng et al., 2018). A rapid rise in cytosolic Ca^2+^ level can be detected within seconds of exposure to salt stress (Jiang et al., 2019). In another hand, salinity inhibited nutrient uptake, including Ca^2+^, just as showed in this paper (Figures 3E,F). However, Ca^2+^ concentrations were inhibited in a higher degree in Adrenalin than Magnum Salt by NaCl. The cyclic nucleotide-gated ion channel (CNGC) family have been implicated in the uptake of cations such as Na^+^, K^+^ and Ca^2+^, so that mediate numerous biological processes ranging from plant development to stress tolerance. Among *CNGC* members in Arabidopsis, *AtCNGC3* helps in seed germination and cation transport, whereas *AtCNGC19* and *AtCNGC20* are associated with salt tolerance (Gobert et al., 2006, Kugler et al., 2009. Both *CNGC3* and *CNGC20* were expressed at higher levels in Magnum Salt than in Adrenalin at 2 h. Autoinhibited Ca^2+^-ATPases (ACAs) can mediate Ca^2+^ efflux to help restore [Ca^2+^]_cyt_ to resting basal levels, for example, AtACA1, 2, and 7 mediate Ca^2+^ efflux from cytoplasm to endoplasmic reticulum and contribute to growth and pollen fitness in a redundant way (Ishka et al., 2021). There were four ACAs that were upregulated by salt. Among them, *ACA2* and *ACA12* involved in Ca^2+^ homeostasis were expressed at higher levels in Magnum Salt at 26 h. It’s interesting to find out whether they are involved in salt tolerance at future.

### Antioxidative System

Oxidative injury happened in about all biotic and abiotic stress as a secondary injury. Thus, the ability to scavenge ROS efficiently is vital for tolerance (Nadarajah, 2020). Salt-sensitive XJ had high levels of ROS and elevation of ROS-related enzyme activity in response to salinity stress, whereas the salt-tolerant ZM showed relatively lower levels of ROS production and unaltered activity of ROS-related enzymes (Lei et al., 2018). Bhattarai et al. (2021) found out that salt tolerance in alfalfa appears to be associated with consistent expression of genes for enhancing defense against oxidative damage and protection against lipid peroxidation. In our research, salinity stress caused accumulation of O_2_^⋅–^ and H_2_O_2_ in alfalfa leaves and upregulation of SOD, CAT, GR and APX activities in both roots and shoots. Magnum Salt accumulated less ROS in leaves than Adrenalin, corresponding with the higher SOD and CAT activities in Magnum Salt than in Adrenalin under salt treatment. Higher levels of SOD, CAT and GR were observed in roots in Magnum Salt than that in Adrenalin (Figures 4A–H and 5). Transcriptional levels of genes encoding antioxidative enzymes were quickly upregulated after salt treatment in Magnum Salt, indicating its fast response to apply antioxidative enzymes to scavenge ROS (Figure 8B and Supplementary Table 5).

### Fatty Acids Metabolization

Membrane fluidity and integrity are largely affected by lipid composition and the degree of fatty acid desaturation in plants (Mikami and Murata, 2003). Alterations in membrane lipids in response to salinity have been observed in a number of plant species including both halophytes and glycophytes (Guo et al., 2019). Membrane fatty acids unsaturation was suggested to affect cell salinity tolerance by affect membrane fluidity so that corelated to Na^+^ and Cl^–^ transport (Lin and Wu, 1996). In peanut, unsaturated fatty acids improved salt tolerance by alleviating photoinhibition (Liu et al., 2017). It has been reported in buffalograss (*Buchloe dactyloides*; Lin and Wu, 1996), corn (*Zea mays* L.; Hajlaoui et al., 2009) and Fabaceae (Bejaoui et al., 2016) that salt-tolerant clones or species had higher unsaturated fatty acids ratio than salt-sensitive ones after salinity treatment. However, no research has investigated the fatty acids contents under salt stress in alfalfa. In our research, it was found that palmitic (16:0), linoleic (18:2) and linolenic acid (18:3) accounted for the largest proportion of the fatty acid in alfalfa (Figure 6A), which was conserved across plant species (Guo et al., 2019). Salinity stress caused a decrease in total unsaturated fatty acids in salt-sensitive Adrenalin, which has been observed in many other plants. However, no significant difference existed in unsaturated fatty acids abundance in Magnum Salt between control or treated group (Figure 6B). Among unsaturated fatty acids, linoleic acid in Magnum Salt increased significantly after 6 days’ salinity. However, no significant difference existed in Adrenalin (Figure 6A).

There are four types of key enzyme in fatty acids synthesis, including fatty acid synthases (FAS), 3-ketoacyl-CoA thiolase (ACAA), β-ketoacyl-coenzyme A (CoA) synthases (KCS) and fatty acid desaturase (FAD). ACAA family members are responsible for the thiolytic cleavage of straight chain 3-oxoacyl-CoAs. KCS mediates the synthesis of very-long-chain fatty acids (VLCFAs) from 22 to 26 carbons in length. Research showed that *KCS1* was salt-inducible, and might play a role in adapting intracellular membrane compartments to function in balancing the external osmotic pressure in extreme halotolerant alga *Dunaliella salina* (Azachi et al., 2002). FAD family members are responsible for fatty acid unsaturation. Endoplasmic reticulum localized FAD2 and FAD6 in plastids convert oleic acid (18:1) to linoleic acid (18:2) by inserting a double bond at the ω-6 position. Whereas FAD3, FAD7, and FAD8 convert linoleic acid (18:2) to linolenic acid (18:3). By investigating the phenotype of mutation materials, FAD2 and FAD6 were proved to be required for salt tolerance probably by maintaining proper function of membrane attached Na^+^/H^+^ exchangers in *Arabidopsis* (Zhang et al., 2012). Lipoxygenase (LOX) is also involved in fatty acid metabolization by catalyzing the hydroperoxidation of linolenic acid. High lipoxygenase activity and its further upregulation by salt stress are the unique features of salt-sensitive sunflower seedlings (Gogna and Bhatla, 2020). In our result, fatty acid metabolization pathway were highly enriched in profile 0 DEGs in Magnum Salt (Supplementary Table 3). Expression levels of *ACAA1*, *ACAA1b*, *KCS1*, *FAD2*, and *FAD7* were upregulated by salinity to higher levels in Magnum Salt than that in Adrenalin, except for *KCS2*, which was highly expressed in Adrenalin. The transcript levels of *LOX1.1* and *LOX1.4* were higher in Adrenalin than Magnum Salt (Figure 8C and Supplementary Table 6). These results might partially explain the higher content of unsaturated fatty acids in Magnum Salt under salt stress.

## Conclusion

**FIGURE 10:**
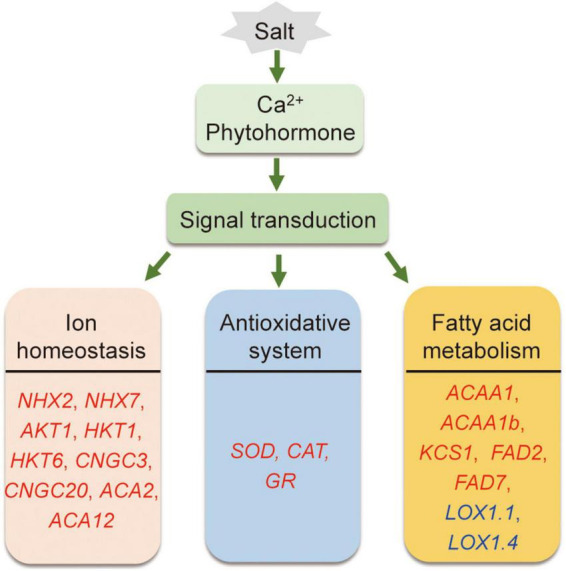
A proposed model showing salt tolerance mechanisms in alfalfa. Salt tolerance in alfalfa is associated with regulation of ionic homeostasis, antioxidative enzymes and fatty acid metabolism. Red and blue genes are proposed positive and negtive regulatory genes in salt tolerance.

In conclusion, compared to Adrenalin, salt-tolerant Magnum Salt applied different strategies to cope with salinity condition by regulation of ionic homeostasis, antioxidative enzyme activities and fatty acid metabolization at both transcriptional and physiological level (Figure 10).

## Data Availability Statement

The datasets presented in this study can be found in online repositories. The names of the repository/repositories and accession number(s) can be found in the article/Supplementary Material.

## Author Contributions

ZG and HZ designed the experiments. JL, MM, YS, and PL performed the experiments. JL, HZ, and HS contributed to the data analysis. HZ and ZG wrote and revised this manuscript. All authors approved the submitted version.

## Conflict of Interest

The authors declare that the research was conducted in the absence of any commercial or financial relationships that could be construed as a potential conflict of interest.

## Publisher’s Note

All claims expressed in this article are solely those of the authors and do not necessarily represent those of their affiliated organizations, or those of the publisher, the editors and the reviewers. Any product that may be evaluated in this article, or claim that may be made by its manufacturer, is not guaranteed or endorsed by the publisher.

## Footnotes

1
http://www.r-project.org/

2
http://systemsbiology.cau.edu.cn/agriGOv2/

3
http://kobas.cbi.pku.edu.cn/
